# Application of Optical Coherence Tomography and Contrast Sensitivity Test for Observing Fundus Changes of Patients With Pregnancy-Induced Hypertension Syndrome

**DOI:** 10.1097/MD.0000000000001641

**Published:** 2015-11-06

**Authors:** Zhixue Wang, Yuanyuan Zou, Wenying Li, Xueyan Wang, Min Zhang, Wenying Wang

**Affiliations:** From the Department of Ophthalmology, Cangzhou Central Hospital, Cangzhou, Hebei, China.

## Abstract

This study was aimed to investigate the fundus changes of patients with pregnancy-induced hypertension syndrome (PIHS) using optical coherence tomography (OCT) technology and contrast sensitivity (CS) tests.

Ninety-eight patients with PIHS underwent routine eye examinations including vision correction, fundus examination, OCT, and CS tests. The CS test was performed at low, medium, and high frequency, respectively. Moreover, the difference in CS tests between 2 groups was analyzed by independent-samples T test. The Kruskal–Wallis rank sum test and linear regression model were used to detect the correlation of OCT with CS, respectively. Meanwhile Satterthwaite approximate T test was adopted for pairwise comparisons after nonparametric analysis of variance.

The OCT test revealed that 56.76% of the examined eyes showed shallow retinal detachment in the macula lutea and around the optic disk. The differences in CS at each spatial frequency between the case and control group were statistically significant (*P* < 0.01). Besides, OCT manifestations were associated with CS at each spacial frequency including 1.5, 3, 6, 12, and 18 frequency (*P* < 0.01). And patients with abnormal manifestations of OCT showed lower CS at each spacial frequency than those without abnormal OCT manifestations. What's more the OCT manifestation 1 showed the greatest impact on CS at each spacial frequency.

The results showed that abnormal OCT manifestations were correlated with CS in PIHS. OCT and CS tests might be valuable methods in observing fundus changes for PIHS patients.

## INTRODUCTION

Hypertensive disorders complicating pregnancy, also known as pregnancy-induced hypertension syndrome (PIHS),^1–3^ is a complication of pregnancy which includes gestational hypertension, preeclampsia, eclampsia, preeclampsia complicated by chronic hypertension and chronic hypertension complicating pregnancy.^4–7^ The pathogenesis and clinical procedures are different between the first 3 diseases and the latter 2, and the present study mainly focuses on the first 3 diseases. Main clinical manifestations of PIHS include hypertension, proteinuria, general dropsy, and an eye disease called retinopathy. Retinal vessels are the only body parts of which the vascular changes can be directly observed in vivo, and fundus examination,^8–10^ was the principal criterion to evaluate the development degree of PIHS in the early years.

Optical coherence tomography (OCT),^11–13^ has found wide application in ophthalmology in recent years. Characteristics such as noncontact, noninvasion, and imaging techniques with high resolution of the OCT can allow people to have a deeper understanding about fundus changes. In the past, progression of eye diseases was usually assessed through examination of visual acuity, which, however, can only evaluate the acuity of eyes in high-contrast environment. Contrast sensitivity (CS), nevertheless, indicates the ability of human eyes to identify visual objects at different spacial frequencies, and thus is a more comprehensive index for visual function estimation. Therefore, we examined the fundus changes of 98 patients with PIHS recruited by Cangzhou Central Hospital using OCT and CS test in the present study.

## OBJECTS AND METHODS

### General Information

The 98 PIHS patients (including 63 primiparas and 35 multiparas) diagnosed by gynecology and obstetrics were proved by eye examinations to have fundus changes, and had no medical histories of chronic hypertension, cardiovascular diseases, diabetes mellitus, and nephrosis. They were aged 23–45 years and had an average age of 29.10 ± 4.88. Their gestational weeks ranged from 21 to 39, with 12 between 20 and 25, 51 between 25 and 30, 18 between 30 and 35, and 17 above 35. When undergoing the eye examination for the first time, they had a mean PIHS course of 10.22 ± 8.60 days and an average gestation of 34.03 ± 3.46 weeks. They also had an average systolic pressure of 178.28 ± 15.57 mm Hg and an average diastolic pressure of 94.34 ± 6.16 mm Hg.

## METHODS

The research was approved by the ethical committee of Cangzhou Central Hospital. Written consents were obtained from all patients. All patients underwent routine eye examinations including vision correction, fundus examination, OCT, and CS tests. OCT test was performed with the HD-OCT4000 (Zeiss Company, Germany) and a long-distance CS test was performed with the OPTEC6500 vision tester (Stereo Company, US) in the daytime under nonglare conditions. The CS of 2 eyes was tested respectively at low frequency (1.5c/d, 3c/d), medium frequency (6c/d), and high frequency (12c/d, 18c/d). The CS was repeatedly measured for 2–3 times at each spacial frequency and average values were automatically calculated by computer analysis softwares. Fifty pregnancy women (100 eyes) with no PIHS, diabetes as well as medical histories of chronic hypertension, cardiovascular diseases, diabetes, nephrosis, organic eye diseases, and systemic diseases affecting eyes were recruited as our control subjects. The controls including 35 primiparas and 15 multiparas had a best corrected visual acuity no lower than 0.8, an average age of 27.12 ± 4.15 (24–42) years, a mean gestation of 35 ± 3.12 weeks, and average systolic and diastolic pressures respectively of 115.33 ± 5.61 and 74.21 ± 5.17 mm Hg. There only existed statistically significant differences in blood pressures between the case and control groups.

### Statistical Analysis

Statistical analysis was accomplished through SPSS 17.0 software. Independent-samples T test was used to analyze the differences in CS tests between the 2 groups. The correlation of OCT with CS was detected using Kruskal–Wallis rank sum test and Satterthwaite approximate test was adopted for pairwise comparisons after nonparametric analysis of variance. Differences had statistical significance when *P* < 0.05. Linear regression model was established to analyze the association between OCT and CS by taking OCT as the independent variable and the corrected visual acuity or CS as the dependent variable.

## RESULTS

### PIHS Diagnostic Criteria

According to *Obstetrics and Gynecology* (the 6th edition), PIHS includes pregnancy-induced hypertension, (mild and severe) preeclampsia and eclampsia.^14^ Among the 98 cases, there were 10 pregnancy-induced hypertension cases (10.21%), 22 mild preeclampsia cases (22.44%), 60 severe preeclampsia cases (61.23%), and 6 eclampsia cases (6.12%).

### OCT Manifestations

**FIGURE 1:**
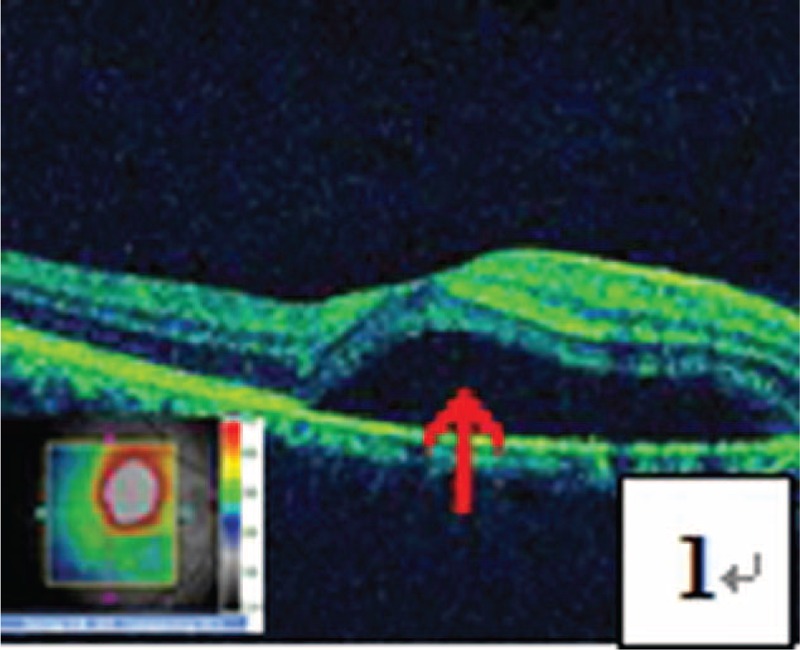
OCT test showed detachment of the retinal neurepithelium layer in the central fovea of macula (pointed by a red arrow).

**FIGURE 2:**
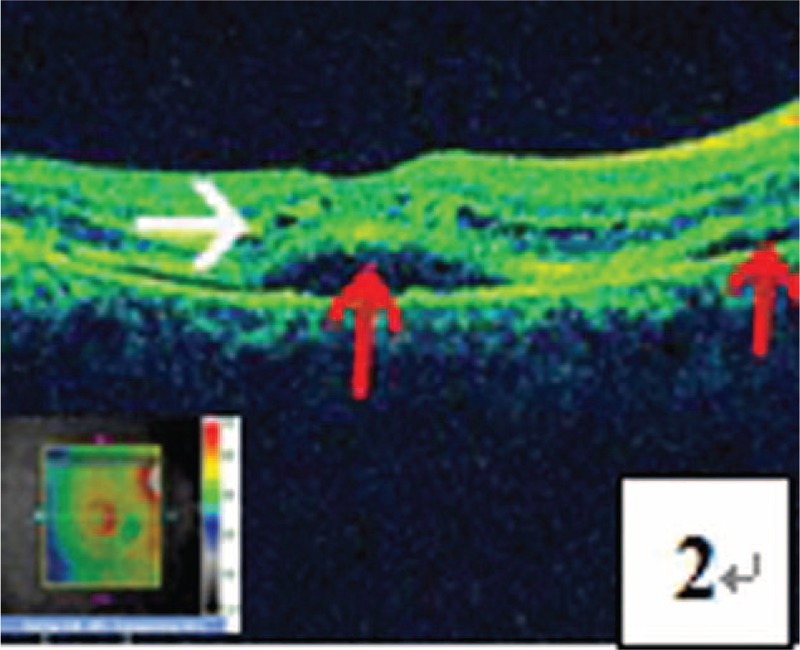
OCT test indicated detachment of the retinal neurepithelium layer in the central fovea of macula and regions on the temporal side of the optic disk (pointed by a red arrow) as well as cystoid edema of the retinal neurepithelium layer (pointed by a white arrow).

**FIGURE 3:**
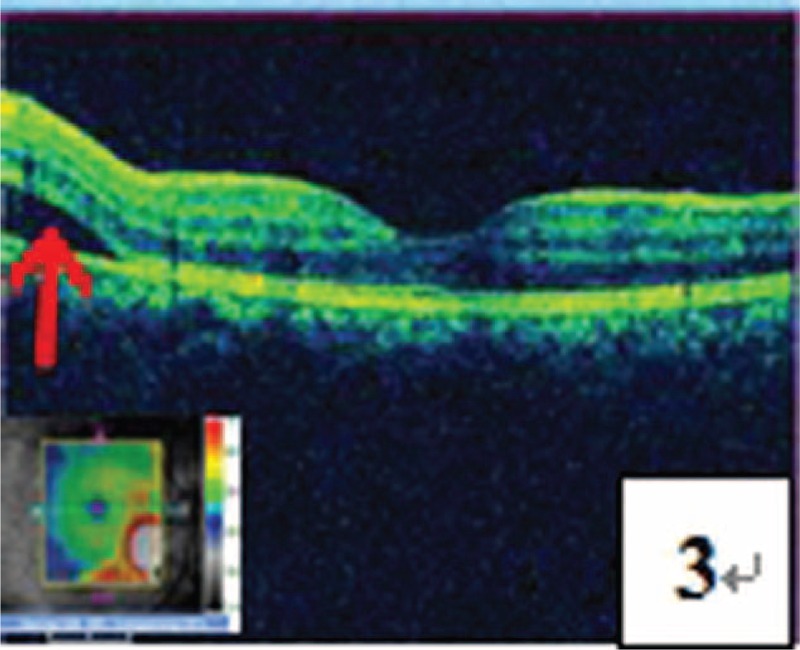
OCT test demonstrated detachment of the retinal neurepithelium layer on the nasal side of the central fovea of macula (pointed by a red arrow), with the central fovea of macula not being involved.

**FIGURE 4:**
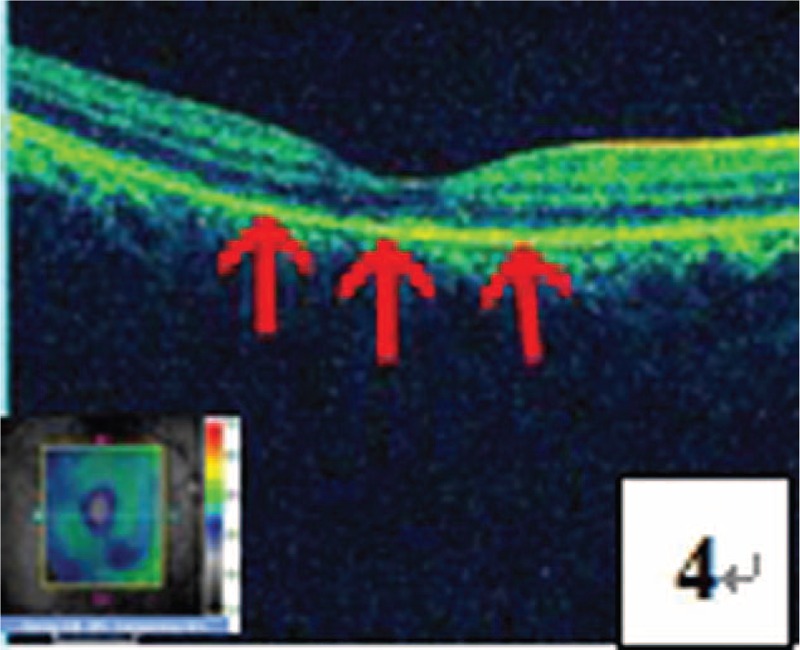
OCT test revealed irregular, discontinuous, and incomplete pigment epithelium layer and ellipsoid layer in the macular region (pointed by a red arrow).

In the first OCT test, 148 eyes were found abnormal among the 196 eyes of the 98 patients (Figures 1–4). Specifically, there were 84 eyes (56.76%) with serous detachment of the retinal neurepithelium layer, including 50 in the central fovea of macula (involving 12 with both serous detachment and edema of the retinal neurepithelium layer) and 34 in other regions of the eye (mainly around the optic disk). Thirty-eight eyes (25.68%) had changes in pigment epithelium layer and ellipsoid layer (formerly called IS/OS layers), and 26 eyes (17.56%) had other changes like optic disc edema and retinal hemorrhage. For the 100 eyes of the 50 pregnancy women in control group, only 2 eyes showed slight changes in pigment epithelium and ellipsoid layer. The differences in OCT manifestations between the 2 groups were with statistical significance (χ^2^ = 145.473, *P* < 0.05).

### CS at Different Spacial Frequencies

The differences in the CS at each spatial frequency between the case and control group were statistically significant (*P* < 0.01) (Table 1).

**TABLE 1:**
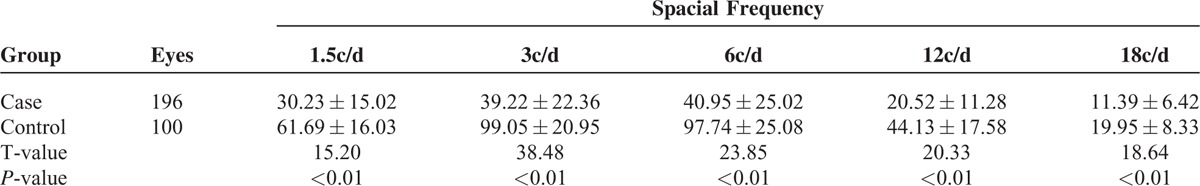
CS at Different Spacial Frequencies of Case and Control Groups

### Investigation of Correlation Between OCT and CS

#### Association Between OCT and CS

Four phenotypic variables 0, 1, 2, and 3 were, respectively, used to represent the following 4 most common OCT manifestations: no obvious abnormality, detachment of the retinal neurepithelium layer, changes in pigment epithelium and ellipsoid layer, and other changes such as optic disc edema and retinal hemorrhage. OCT manifestations were associated with CS at each spacial frequency with *P* < 0.01 (Table 2). Pairwise comparisons demonstrated that the differences between variables 2 and 3 in the CS at any of the spacial frequencies were not significant, but the differences in the CS at any of the spacial frequencies between other groups had statistical significance (Table 3).

**TABLE 2:**
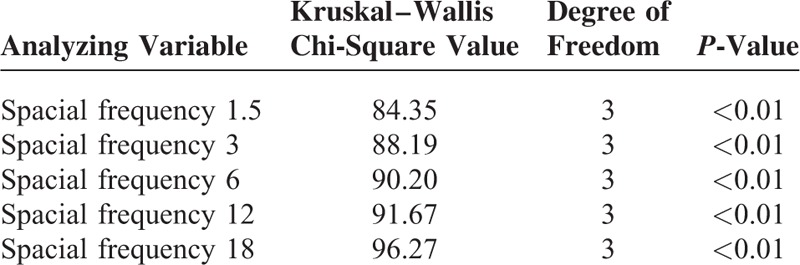
Correlation of OCT With CS

**TABLE 3:**
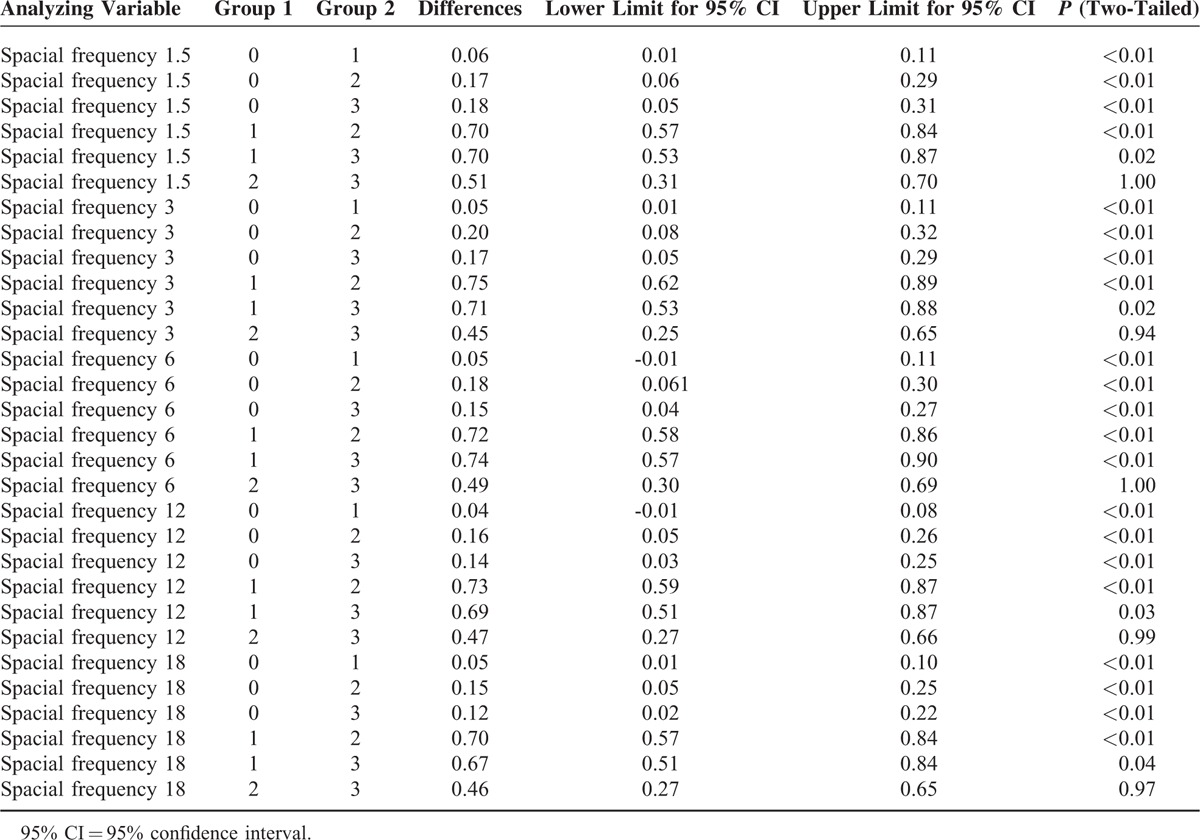
Pairwise Comparisons of OCT Manifestations Under Different CS

### Establishment of Linear Regression Models

To study the association between OCT manifestations and CS, linear regression models spacial frequency∼ variable (OCT) were established, with OCT manifestation being the independent variable and CS being the dependent variable.

In the linear regression model spacial frequency 1.5∼ variable (OCT), the outcome variable was spacial frequency 1.5 and the variable distribution and linkage was analyzed by Gaussian distribution function. As shown in Table 4, regression coefficients between the 3 OCT manifestations and CS at spacial frequency 1.5 were respectively −25.67, −18.77, −18.84 (*P* < 0.01), indicating that the 3 abnormal OCT manifestations were risk factors for the decreasing of CS at spacial frequency 1.5 and patients with the 3 abnormal OCT manifestations showed lower CS at spacial frequency 1.5 than those without abnormal OCT manifestations. Moreover, OCT manifestation 1 had the biggest influences on the CS at 1.5 spacial frequency.

**TABLE 4:**
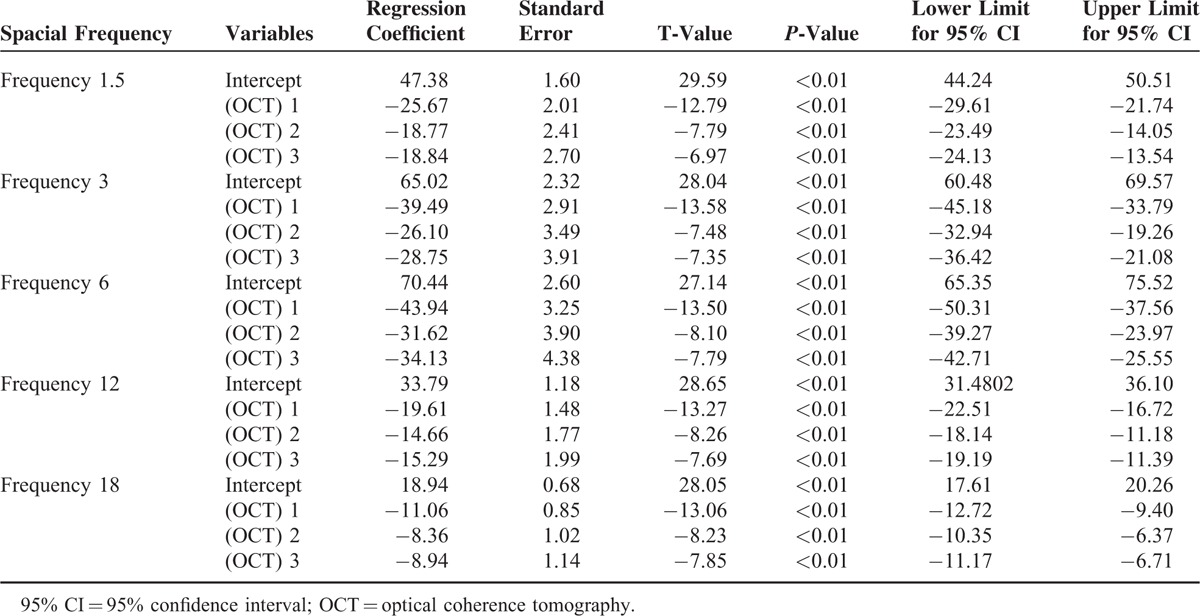
Association Between OCT Manifestations and CS at Different Spacial Frequency

In the linear regression model spacial frequency 3∼ variable (OCT), the outcome variable was spacial frequency 3 and Gaussian distribution function was used to analyze the variable distribution and linkage. It could be seen from Table 4 that the regression coefficients between the 3 OCT manifestations and CS at spacial frequency 3 were, respectively, −39.49, −26.10, −28.75 (*P* < 0.01), which showed that the 3 abnormal manifestations of OCT could cause reduced CS at spacial frequency 3, and the CS at spacial frequency 3 was lower for patients with the 3 abnormal OCT manifestations than for those without the manifestations. What's more, the CS at spacial frequency 3 was most significantly influenced by OCT manifestation 1.

In the linear regression model spacial frequency 6∼ variable (OCT), the outcome variable was spacial frequency 6, and the Gaussian distribution function was adopted for analyzing of variable distribution and linkage. Table 4 shows that regression coefficients were, respectively, −43.94, −31.62, −34.13 (*P* < 0.01) between 3 OCT manifestations and CS at spacial frequency 6, demonstrating that the 3 abnormal manifestations of OCT could contribute to decreased CS at spacial frequency 6, and patients with no abnormal OCT manifestations had higher CS at spacial frequency 6 than those with abnormal OCT manifestations. Additionally, the biggest influences on CS at spacial frequency 6 were from OCT manifestation 1.

In the linear regression model spacial frequency 12∼ variable (OCT), the outcome variable was spacial frequency 12, and Gaussian distribution function was applied for assessing of variable distribution and linkage. As demonstrated in Table 4, −19.61, −14.66, −15.29 were, respectively, the regression coefficients between the 3 manifestations of OCT and CS at spacial frequency 12 (*P* < 0.01). That meant that the 3 abnormal manifestations of OCT were related to the decreased CS at spacial frequency 12. Patients with the 3 abnormal OCT manifestations displayed lower CS at spacial frequency 12 than those without abnormal OCT manifestations. In addition, the OCT manifestation 1 exerted the greatest impact on CS at spacial frequency 12.

In the linear regression model spacial frequency 18∼ variable (OCT), the outcome variable was spacial frequency 18, and variable distribution and linkage was evaluated using Gaussian distribution function. As manifested in Table 4, the regression coefficients were, respectively, −11.06, −8.36, −8.94 (*P* < 0.01) between the 3 manifestations of OCT and CS at spacial frequency 18, displaying that associations existed between the 3 abnormal manifestations of OCT and reduced CS at spacial frequency 18. Patients without the 3 OCT manifestations demonstrated higher CS at spacial frequency 18 than those with abnormal OCT phenotypes. The OCT manifestation 1 showed the greatest impact on CS at spacial frequency 18.

## DISCUSSION

PIHS usually occurs during late pregnancy. According to clinical experience, patients who have dropped blood pressure after such measures as resting, taking no salt, as well as using analgesics and hypotensor may continue the pregnancy, during which period frequent checking of the fundus is required. If the drug treatment were ineffective and retinal edema, exudation or hemorrhage occurred, it would be indicated that organic lesions had occurred in the retinas and arteriolae of the whole body. In this case, termination of pregnancy should be timely performed to avoid severe complications. Therefore, fundus changes, especially serious retinopathy may be important criteria for termination of pregnancy.^15^ Ophthalmofundoscopy may cause missed diagnosis of such severe retinopathy as retinal edema and shallow retinal detachment in some patients. Even ultrasound examination may fail to diagnose shallow retinal detachment, and it is inappropriate to check fundus changes of pregnant women with fundus fluorescence angiography (FFA),^16–18^ due to the use of contrast media.^19^ However, the nonintrusive and repeatable OCT test allows high-resolution cross-sectional microimaging of ocular structures, which can facilitate observations of the retina and other nearby tissues and thus provide objective and dependable basis for clinical diagnosis and treatment.

The OCT test results of this study demonstrated that 56.76% of the examined eyes showed shallow retinal detachment in the macula lutea and around the optic disk. Researchers have found that PIHS patients may suffer from varying degrees of delayed choroidal vascular filling or choroidal nonperfusion in early phase of the disease, which are manifestations of blood circulation disorder and ischemia in the choroid and can be shown by less or no fluorescence observed in FFA.^20^ In ischemic regions, pigment epithelium changes such as blocked fluorescence and fluorescence leakage can be observed; and this phenomenon usually occurs around the optic disk and in macular area. All these imply that pigment epithelium damages caused by choroid ischemia may lead to barrier dysfunctions of the choroid membrane, and fluid leakage under the retina can then result in retinal detachment, which can be properly shown by OCT. The present study also suggested that retinal changes in morphology, especially shallow retinal detachment and tiny changes of ellipsoid layer and pigment epithelium, of PIHS patients could be distinctly observed via OCT test.

Visual CS is a criterion for form sense inspection. It refers to the ability of the visual system to recognize the sinusoidal grating of different spacial frequencies under different brightness contrasts, and is the black-and-white contrast (contrast ratio) of the object surface that is needed by the visual system to identify the spacial frequencies (c/d) of objects with different sizes. In the present study, the visual targets for sine wave stripes were bright-dark stripes, 1 pair of which meant 1 circle; and spacial frequency (the number of circles per view angle degree (c/d)) represented the thickness of the stripes, with inverse correlation existing between the stripe thickness and the spatial frequency. Each spacial frequency has a contrast threshold, which is the minimum contrast distinguished by human eyes. When the spatial frequency is lower than that threshold, stripes would turn to a uniform gray color which cannot be distinguished by human eyes. Reciprocal value of the contrast threshold is the CS. Stripes show the most clear borders when the contrast ratio is 100% (the maximum), which is the case when visual chart is used for conventional examination of visual acuity.

However, optic examination with visual chart can only reflect the function of the macula lutea to identity small targets under high contrast, which is not always the case in normal life, so it can only assess the visual function very simply and gives far less physiological and pathological information about the visual sense than the CS test.^21^ In contrast, the CS test evaluates the visual function by changing 2 parameters (spacial frequency and contrast) simultaneously, which can better conform to the practical visual environment for human eyes. For the CS test, good visual functions not only mean that the central vision reaches or approaches 1.0, but also indicate that the CS of the visual system at different spacial frequencies reaches normal levels.^20^ Therefore, the CS test can estimate the vision of the patients more comprehensively and effectively. The results of the present study suggested that the CS at each spacial frequency for the PIHS patients was remarkably lower than that for normal controls, abnormal OCT manifestations were correlated with CS, and the OCT manifestation 1 (retinal detachment) had the most significant influences on the CS at each spacial frequency.

Thus, it is crucial to do regular OCT test simultaneously with prenatal examinations for pregnant women who have a pregnancy duration of more than 20 weeks and a medical history of hypertension or unstable blood pressure. This can contribute to earlier finding of fundus changes, and thus provide diagnostic basis for obstetricians to take medical measures in time. Meanwhile, CS test can better reflect the visual conditions than the conventional optic examination with visual chart. Further studies are needed to confirm the practical value of OCT and CS tests in observing fundus changes of PIHS patients.
